# The Effect of Cigarette Smoke Exposure on the Development of Inflammation in Lungs, Gut and Joints of TNF^ΔARE^ Mice

**DOI:** 10.1371/journal.pone.0141570

**Published:** 2015-11-02

**Authors:** Liesbeth Allais, Smitha Kumar, Karlijn Debusschere, Stephanie Verschuere, Tania Maes, Rebecca De Smet, Griet Conickx, Martine De Vos, Debby Laukens, Guy F. Joos, Guy G. Brusselle, Dirk Elewaut, Claude A. Cuvelier, Ken R. Bracke

**Affiliations:** 1 Department of Medical and Forensic Pathology, Ghent University, Ghent, Belgium; 2 Laboratory for Translational Research in Obstructive Pulmonary diseases, Department of Respiratory Medicine, Ghent University Hospital, Ghent, Belgium; 3 Laboratory for Molecular Immunology and Inflammation, Department of Rheumatology, Ghent University, Ghent, Belgium; 4 Department of Pathology, AZ Sint-Jan, Brugge, Belgium; 5 Department of Gastroenterology, Ghent University, Ghent, Belgium; 6 VIB Inflammation Research Center, Ghent University, Ghent, Belgium; Public Health Research Institute at RBHS, UNITED STATES

## Abstract

The inflammatory cytokine TNF-α is a central mediator in many immune-mediated diseases, such as Crohn’s disease (CD), spondyloarthritis (SpA) and chronic obstructive pulmonary disease (COPD). Epidemiologic studies have shown that cigarette smoking (CS) is a prominent common risk factor in these TNF-dependent diseases. We exposed TNF^ΔARE^ mice; in which a systemic TNF-α overexpression leads to the development of inflammation; to 2 or 4 weeks of air or CS. We investigated the effect of deregulated TNF expression on CS-induced pulmonary inflammation and the effect of CS exposure on the initiation and progression of gut and joint inflammation. Upon 2 weeks of CS exposure, inflammation in lungs of TNF^ΔARE^ mice was significantly aggravated. However, upon 4 weeks of CS-exposure, this aggravation was no longer observed. TNF^ΔARE^ mice have no increases in CD4+ and CD8+ T cells and a diminished neutrophil response in the lungs after 4 weeks of CS exposure. In the gut and joints of TNF^ΔARE^ mice, 2 or 4 weeks of CS exposure did not modulate the development of inflammation. In conclusion, CS exposure does not modulate gut and joint inflammation in TNF^ΔARE^ mice. The lung responses towards CS in TNF^ΔARE^ mice however depend on the duration of CS exposure.

## Introduction

Aberrant cytokine profiles have been linked to several immune-mediated diseases, such as Crohn’s disease (CD), spondyloarthritis (SpA) and chronic obstructive pulmonary disease (COPD) [1,2,3]. TNF-α, a prominent pro-inflammatory cytokine, appears to be an important pathogenic factor in the development of these diseases and is known to mediate a wide range of biological activities [4,5]. TNF-α expression is elevated in affected mucosal areas of inflammatory bowel disease (IBD) patients and in the active disease regions in animal models of intestinal inflammation [6,7,8]. Anti-TNF-α therapy is commonly used as a treatment for CD and SpA [9,10,11]. Levels of TNF-α are increased in sputum, bronchoalveolar lavage (BAL) fluid and serum of patients with COPD, which may further amplify the existing pulmonary inflammation and play a role in the systemic manifestations occurring in a subgroup of COPD patients [12,13]. Although several trials using anti-TNF-α blockers in patients with COPD have revealed no benefits, a large observational study reports reduced hospitalization rates in patients diagnosed with both COPD and rheumatoid arthritis (RA) and receiving TNF-α inhibitors [14].

A prominent risk factor for the development of these TNF-α -mediated diseases is cigarette smoking (CS)[15,16]. CS exerts a detrimental effect on multiple organ systems and adversely affects immune function, promoting the progression of several diseases, such as COPD [17], CD and coronary heart disease [18,19]. Importantly, CS is associated with an increased susceptibility and altered disease course in several auto-immune diseases, including Graves’ disease, multiple sclerosis, SpA, RA and systemic lupus erythematosus [20,21,22,23].

Many studies have focused on the effect of CS on the individual entities (CD, SpA and COPD), however, little is known on the link between the pathologies and the role of CS therein. CD and SpA are clearly linked on a genetic and clinical level. SpA patients are prone to develop CD and vice versa [24,25,26,27]. Mutations in NOD2, IL-23 and HLA-B27 have been linked to both CD and SpA [28,29,30]. CS is the most prominent environmental risk factor for developing CD [31,32]. In addition, the interaction between CS and certain genetic factors, such as the HLA-DR locus, increases the risk for development of arthritis [33].

The well-known TNF^ΔARE^ mouse model, in which the AU-region of the TNF-α mRNA is deleted, results in systemic TNF-α overproduction and is hallmarked by the spontaneous development of chronic Crohn-like ileitis and inflammatory arthritis [34]. To study the effect of deregulated TNF-α expression on CS-induced pulmonary inflammation and the effect of CS exposure on TNF-α-induced gut and joint inflammation, we exposed TNF^ΔARE^ to air or CS for 2 or 4 weeks. Our first hypothesis stated that CS-induced inflammation might aggravate in the lungs of TNF^ΔARE^ mice. We secondly hypothesized that CS might aggravate TNF-induced gut and joint inflammation in TNF^ΔARE^ mice. Inflammation and pro-inflammatory cytokine production was simultaneously investigated in lungs, gut and joints.

## Materials and Methods

### Experimental Mouse Model

C57BL/6 mice heterozygous for tumor necrosis factor (TNF)ΔAU-rich element (ARE) (TNF^ΔARE^) and WT littermates were bred at the animal breeding facility of the Faculty of Medicine and Health Sciences, Ghent University. Mice were housed in filtertop cages in groups of 5 mice per cage, containing untreated wood shavings and nestlets for environmental enrichment (Carfil Quality, Turnhout, Belgium). By 8 weeks of age, TNF^ΔARE^ mice develop disease that worsens progressively. All mice were given free access to chlorinated tap water and offered a standard chow diet *ad libitum* (Carfil Quality, Turnhout, Belgium). The mice (all males) were 6 weeks old at the start of the smoke exposure. The local Ethics Committee for animal experimentation of the Faculty of Medicine and Health Sciences (Ghent, Belgium) approved all experiments (ECD 25/11). The experiments were carried out in accordance with the approved guidelines of the Faculty of Medicine and Health Sciences (Ghent, Belgium).

### Cigarette Smoke Exposure

Mice were exposed to main stream cigarette smoke, as described previously [35]. Groups of 6 to 10 mice were put in a plexiglass chamber connected to a smoking apparatus and exposed to the tobacco smoke of 5 simultaneously lit cigarettes (Reference Cigarette 3R4F without filter; University of Kentucky, Lexington, KY, USA). This exposure was repeated 4 times a day with 30 min. smoke-free intervals, 5 days per week for 2 or 4 weeks. The smoking chamber is fitted with an air supply whereby an optimal smoke:air ratio of 1:6 is obtained. The control groups were exposed to air. Using this protocol, carboxyhemoglobin levels in serum of CS-exposed mice reached 8.35 ± 0.46% (versus 0.65 ± 0.25% in air-exposed mice), corresponding to serum levels in human smokers [36]. Given that full-blown ileal inflammation in TNF^ΔARE^ mice occurs already at 12–14 weeks of age, we decided to start the CS exposure at the early age of 6 weeks. A longer CS exposure than 4 weeks appeared impossible due to ethical reasons.

### Evaluation of clinical parameters, fecal lipocalin-2 (LCN-2) and serum TNF-α and IL-6 levels

The mice were weighed, randomized for weight and scored for arthritis before the start of the first smoke exposure. During the experiment, the mice were weighed twice a week. Fecal samples were collected twice a week and frozen at -80°C until analysis. Clinical scoring for arthritis was performed 3 times a week during the 2-week experiment and twice a week during the 4-week experiment. Mice were bled once a week in both experiments and sera were stored at -20°C.

For quantification of LCN-2 by ELISA, frozen fecal samples were reconstituted in PBS containing 0.1% Tween20 and vortexed for 20 min to get a homogenous fecal suspension as described previously [37]. Subsequently, the samples were centrifuged for 10 min at 12.000 rpm and 4°C. Clear supernatants were collected and stored at -20°C until analysis. LCN-2 levels were measured using the Duoset murine LCN-2 ELISA kit (R&D Systems, Abingdon, Oxon, UK).

TNF-α and IL-6 levels in serum were measured by means of a commercially available enzyme-linked immunosorbent assay (ELISA) for mouse TNF-α and IL-6 (eBioScience, Vienna, Austria).

### Sampling procedure

Mice were weighed and euthanized with an overdose of pentobarbital (Sanofi-Ceva, Paris, France) 24 hours after the last smoke exposure. The peritoneal cavity was opened to take samples of gut. First, ileum sections and Peyer’s patches were recovered. Next, bronchoalveolar lavage (BAL) fluid was collected and samples of the lung were taken. BAL fluid recovery and differential cell counts were performed as described previously [38,39]. Thirdly, the joints of the knee and ankle were sampled.

### Bronchoalveolar lavage (BAL) fluid analysis

Flow cytometric analysis of BAL cells was performed to enumerate dendritic cells (DCs), macrophages, neutrophils and CD4+ and CD8+ T cells. BAL cells were labeled with fluorochrome-conjugated monoclonal antibodies against the following cell markers: CD11c, MHC class II (MHC-II), CD11b, Ly6C, Ly6G, CD3, CD4 and CD8 (BD Pharmigen, San Jose, CA, USA). 7-aminoactinomycin D (BD Pharmigen, San Jose, CA, USA) was used for dead cell exclusion. Data acquisition was performed on a FACSCalibur flow cytometer running Cell Quest software. FlowJo software (Tree star Inc., Ashland, Oregon, USA) was used for data analysis. TNF-α protein level in BAL fluid was determined using a commercially available ELISA kit (eBioScience, Vienna, Austria).

### Hematoxylin and eosin staining and quantification of inflammation in gut and joints

The gut was dissected, terminal ileum was sampled and fixed in phosphate-buffered formalin (pH 7.4) and embedded in paraffin wax. Paraffin-embedded 4 μm tissue sections taken from terminal ileum were dewaxed and rehydrated. The sections were stained with haematoxylin and eosin (H&E), using the Tissue-Tek Prisma/Film automated slide stainer (Sakura, Torrance, US). The degree of ileal inflammation was scored in a blinded manner and independently by two pathologists according to a scoring scheme (Table 1) adapted from Kontoyiannis et al., 2002 [40]. The histologic scoring was performed on the most terminal ileal sections.

**Table 1:** Histologic score to quantify the degree of ileal inflammation.

**Score**	**Acute inflammation**
**0**	0–1 PMN/hpf within mucosa
**1**	2–10 PMN/hpf within mucosa
**2**	11–20 PMN/hpf within mucosa
**3**	21–30 PMN/hpf within mucosa or 11–20 PMN/hpf with extension below muscularis mucosae
**4**	>30 PMN/hpf within mucosa or >20 PMN/hpf with extension below muscularis mucosae
**Score**	**Chronic inflammation**
**0**	0–10 ML/hpf within mucosa
**1**	11–20 ML /hpf within mucosa
**2**	21–30 ML /hpf within mucosa or 11–20 with extension below muscularis mucosae/follicular hyperplasia
**3**	31–40 ML /hpf within mucosa or 21–30 with extension below muscularis mucosae/follicular hyperplasia
**4**	>40 ML /hpf within mucosa or >30 with extension below muscularis mucosae/follicular hyperplasia

PMN: polymorphonuclear cells. ML: mononuclear leukocytes. Hpf: high power field. Scoring scheme adapted from Kontoyiannis et al, 2002.

Total ankle and knee joints were dissected, fixed in phosphate-buffered formalin (pH 7.4), decalcified in 5% buffered formic acid and embedded in paraffin wax. Paraffin-embedded 7 μm tissue sections taken from the joints were dewaxed and rehydrated. H&E staining on the tissue sections was performed using the Tissue-Tek Prisma/Film automated slide stainer (Sakura, Torrance, US). The degree of joint inflammation was evaluated by two blinded assessors. Tarsal and metatarsal joints (ankle) were scored for three parameters: infiltrate in the Achilles tendon and synovio-entheseal complex, calcaneal erosion and exudate at the synovio–entheseal complex and metatarsal joints, each ranging from 0 (normal) to 3. A composite score was built from these parameters resulting in a score ranging up to 6 (adapted from proof of concept by Jacques et al.)[26]. For the knee joints, a composite score was built from three other parameters: synovial infiltrate, exudate in the joint space, cartilage and bone destruction.

### CCL20 immunohistochemistry in terminal ileum

Immunohistochemistry for CCL20 was performed as described by Verschuere et al [41]. Briefly, cryosections of terminal ileum were air-dried and fixed with ice-cold acetone. Endogenous peroxidase activity was quenched with 1% H_2_O_2_, followed by blocking of non-specific binding sites with 2% rabbit serum and 1% BSA in PBS. Subsequently, slides were incubated with the primary antibody (polyclonal goat anti-mouse CCL20, R&D Systems) or goat IgG isotype control (Santa Cruz Biotechnology, Santa Cruz, CA, USA) for 90 min at 37°C and with the biotinylated rabbit anti-goat secondary antibody (DAKO) for 30 min at room temperature. Then, HRP-conjugated streptavidin (DAKO) was applied for 30 min. DAB was used as enzyme substrate before counterstaining with haematoxilin.

### Quantitative real-time PCR

RNA from ileum, Peyer’s patches and lung was extracted using the Qiagen miRNeasy Mini Kit (Qiagen, Hilden, Germany). Subsequently, cDNA was synthesized by reverse transcription using the iScript^™^ cDNA Synthesis kit (Bio-Rad Laboratories, Nazareth, Belgium) following the manufacturer’s instructions. cDNA from total lung RNA was prepared using Transcriptor First strand cDNA synthesis kit (Roche Diagnostics, Basel, Switzerland).

In lungs, expression of target genes Tnf-α, Tnfr1 and Tnfr2, Cxcl1/Kc, Ccl2/Mcp-1, Cxcl2/Mip-2 and reference genes Hypoxanthine-guanine phosphoribosyltransferase (Hprt1), Glyceraldehyde-3-phosphate dehydrogenase (Gapdh) and Transferrin receptor (Tfrc) was measured using TaqMan Gene Expression assays (Applied BioSystems, Forster City, CA, USA). Quantitative real-time PCR was performed in duplicate using 10 ng cDNA with a LightCycler96 detection system (Roche Diagnostics). Expression levels were calculated using a standard curve. Expression of target genes was normalized based on the three reference genes.

For gut, expression of target genes Cxcl1/Kc, Cxcl2, Il-1β, Tnf-α, Ccr1, Ccr6, Ccl20, Atg5, Atg7, Beclin-1 and Atg16L1 (sequences are provided in Table 2), was analyzed by qRT-PCR using the SensiMix^™^ SYBR No-ROX Kit (Bioline, London, UK). As reference genes, Succinate dehydrogenase (Sdha) and Hydroxymethylbilane synthase (Hmbs) were used to normalize ileal gene expression. qRT-PCR was performed on a LightCycler480 detection system (Roche, Vilvoorde, Belgium) with the following cycling conditions: 10 min incubation at 95°C, 45 cycles of 95°C for 10 seconds and 60°C for 1 min. Melting curve analysis confirmed primer specificity. The PCR efficiency of each primer pair was calculated using a standard curve from reference cDNA. The amplification efficiency was determined using the formula 10^−1/SLOPE^ - 1.

**Table 2:** Mouse primer sequences qRT-PCR.

Gene Symbol	Accession Number	Forward Primer (5’-3’)	Reverse primer (3’-5’)	Effic	R^2^
Hmbs	NM_001110251	AAGGGCTTTTCTGAGGCACC	AGTTGCCCATCTTTCATCACTG	99	0,99
Hprt	NM_013556	GTTAAGCAGTACAGCCCCAAA	AGGGCATATCCAACAACAAACTT	95	0,9974
Sdha	NM_023281	CTTGAATGAGGCTGACTGTG	ATCACATAAGCTGGTCCTGT	97	0,9981
Cxcl1/Kc	NM_008176	ACCGAAGTCATAGCCACACTC	TCTCCGTTACTTGGGGACAC	95	0,9995
Cxcl2	NM_009140	GCGCCCAGACAGAAGTCATAG	AGCCTTGCCTTTGTTCAGTATC	89,2	0,99
Il-1β	NM_000576	CACGATGCACCTGTACGATCA	GTTGCTCCATATCCTGTCCCT	97	0,9987
Tnf-α	NM_013693	ATGAGCACTGAAAGCATGATCC	GAGGGCTGATTAGAGAGAGGTC	92	0,9761
Ccr1	NM_009912	CTCATGCAGCATAGGAGGCTT	ACATGGCATCACCAAAAATCCA	93	0,8432
Ccr6	NM_009835	CCTGGGCAACATTATGGTGGT	CAGAACGGTAGGGTGAGGACA	115	0,9824
Ccl2	NM_011333	GCATCTGCCCTAAGGTCTTCA	TGCTTGAGGTGGTTGTGGAA	107	0,9958
Ccl9	NM_011338	CCCTCTCCTTCCTCATTCTTACA	AGTCTTGAAAGCCCATGTGAAA	106	0,992
Ccl19	NM_011888	GGGGTGCTAATGATGCGGAA	CCTTAGTGTGGTGAACACAACA	89	0,928
Ccl20	NM_016960	GCCTCTCGTACATACAGACGC	CCAGTTCTGCTTTGGATCAGC	95	0,9991
Atg5	NM_053069	TGTGCTTCGAGATGTGTGGTT	ACCAACGTCAAATAGCTGACTC	98	0,9985
Atg7	NM_028835	TCTGGGAAGCCATAAAGTCAGG	GCGAAGGTCAGGAGCAGAA	105	0,9901
Atg16l1	NM_001205392	CAGAGCAGCTACTAAGCGACT	AAAAGGGGAGATTCGGACAGA	96	0,9901
Beclin-1	NM_019584	ATGGAGGGGTCTAAGGCGTC	TGGGCTGTGGTAAGTAATGGA	107	0,9912

### Statistical analysis

Reported values are expressed as mean ± standard error of the mean (SEM). Statistical analysis was performed by SPSS 21 Software (SPSS 21 Inc., Chicago, IL, USA) using Student’s t-test for normally distributed populations and Kruskal Wallis or Mann–Whitney U-test for populations where normal distribution was not accomplished. Mixed model analysis was performed for multiple time points. A p-value of less than 0.05 was considered significant.

## Results

### Cigarette smoke-induced pulmonary inflammation in TNF^ΔARE^ mice is aggravated upon 2 weeks, but not upon 4 weeks of CS exposure

**Fig 1:**
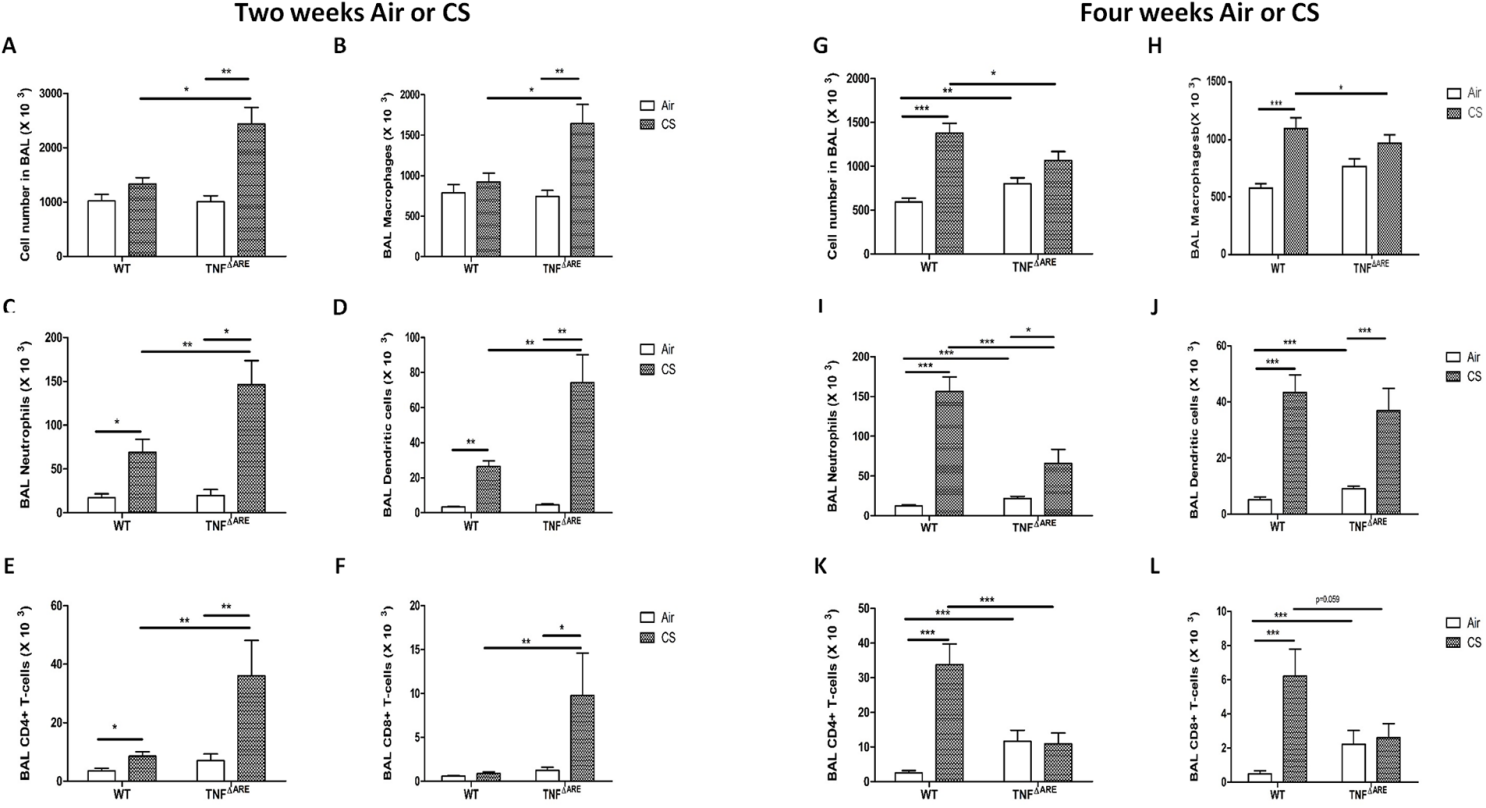
Inflammatory cell counts in BAL fluid of WT and TNF^ΔARE^ mice. Numbers of **(A)** total cells, **(B)** macrophages, **(C)** neutrophils, **(D)** dendritic cells, **(E)** CD4^+^ T-lymphocytes and **(F)** CD8^+^ T-lymphocytes in BAL fluid of mice exposed to air or CS during 2 weeks. Numbers of **(G)** total cells, **(H)** macrophages, **(I)** neutrophils, **(J)** dendritic cells, **(K)** CD4^+^ T-lymphocytes and **(L)** CD8^+^ T-lymphocytes in BAL fluid of mice exposed to air or CS during 4 weeks. Values are expressed as mean ± SEM. * p<0.05, ** p<0.01, *** p<0.001.

To evaluate the effect of cigarette smoke (CS) exposure on the lungs of TNF^ΔARE^ mice, we measured inflammatory cells in BAL fluid of WT and TNF^ΔARE^ mice exposed to air or CS for 2 and 4 weeks. WT mice exposed to CS for 2 weeks showed a modest, but significant increase in the absolute numbers of neutrophils, dendritic cells (DC) and CD4+ T-lymphocytes compared to air-exposed control mice (Fig 1C–1E). Interestingly, in TNF^ΔARE^ mice, the CS-induced inflammation in BAL was significantly aggravated compared to the WT mice (Fig 1A–1F), resulting in significantly increased numbers of macrophages, neutrophils, DCs and CD4+ and CD8+ T lymphocytes. Upon 4 weeks of CS exposure, the WT mice showed further increase in the number of inflammatory cells compared to 2 weeks of CS exposure (Fig 1H–1L). However, the aggravated pulmonary inflammatory response in TNF^ΔARE^ mice was no longer observed after 4 weeks of CS exposure, and even significantly reduced levels of neutrophils and CD4+ T cells could be detected.

**Fig 2:**
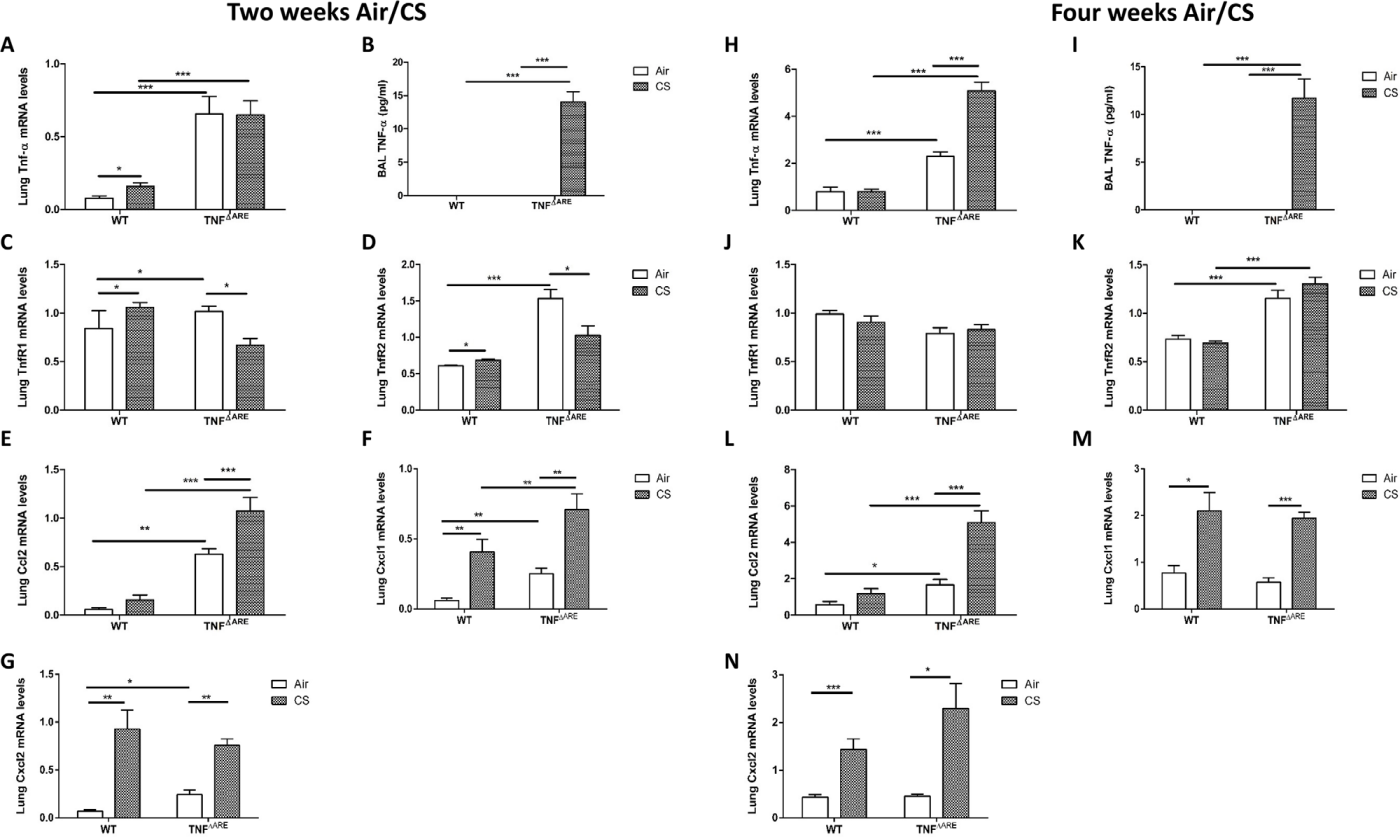
Inflammatory gene expression in lung tissue and BAL fluid of WT and TNF^ΔARE^ mice. mRNA expression of **(A)** Tnf-α, **(C)** Tnfr1, **(D)** Tnfr2, **(E)** Ccl2, **(F)** Cxcl1 and **(G)** Cxcl2 in lung tissue of WT and TNF^ΔARE^ mice exposed to air or CS during 2 weeks. **(B)** Protein levels of TNF-α in BAL fluid of WT and TNF^ΔARE^ mice exposed to air or CS during 2 weeks. mRNA expression of **(H)** Tnf-α, **(J)** Tnfr1, **(K)** Tnfr2, **(L)** Ccl2, **(M)** Cxcl1 and **(N)** Cxcl2 in lung tissue of WT and TNF^ΔARE^ mice exposed to air or CS during 4 weeks. **(I)** Protein levels of TNF-α in BAL fluid of WT and TNF^ΔARE^ mice exposed to air or CS during 4 weeks. Expression levels are relative to the expression of 3 reference genes (hypoxanthine phosphoribosyltransferase 1 (Hprt1), glyceraldehyde-3-phosphate dehydrogenase (GAPDH) and transferring receptor protein 1 (Tfrc)). Values are expressed as mean ± SEM. * p<0.05, ** p<0.01, *** p<0.001.

The mRNA expression level of TNF-α was higher in the lungs of TNF^ΔARE^ mice compared to WT mice and significantly increased after 4 weeks of CS, but not after 2 weeks. Also, CS exposure for 2 and 4 weeks significantly increased TNF-α protein levels in BAL fluid of TNF^ΔARE^ mice (Fig 2A, 2B, 2H and 2I). Consequently, we examined the expression of the TNF-α receptors. The expression of TNFR1 and TNFR2 is differentially affected by CS depending on duration of exposure (Fig 2C, 2D, 2J and 2K). Two weeks of CS exposure significantly increased TNFR1 and TNFR2 gene expression levels in lung tissue of WT mice, while the expression of both receptors decreased in TNF^ΔARE^ mice. Upon 4 weeks CS exposure, no difference in TNFR1 and TNFR2 mRNA levels could be observed between air- and CS-exposed WT or TNF^ΔARE^ mice.

In line with the increased inflammatory cells, gene expression levels of CXCL1, CXCL2 and CCL2 were higher in lungs of TNF^ΔARE^ mice compared with WT mice exposed to CS for 2 weeks. Although the inflammatory cell response decreased after 4 weeks CS exposure, gene expression levels of inflammatory mediators remained elevated in lungs of TNF^ΔARE^ mice (Fig 2E–2G and 2L–2N).

### CS exposure does not affect macroscopic clinical parameters of ileitis, ileal histology, fecal lipocalin-2 levels and inflammatory and autophagy gene expression

**Fig 3:**
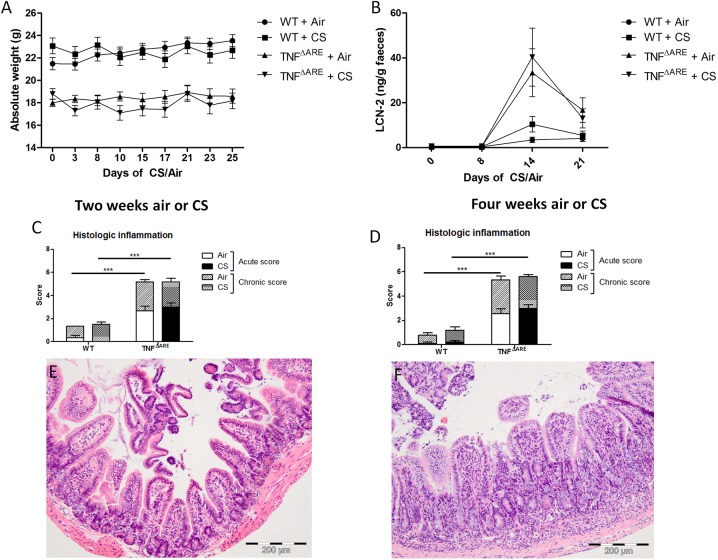
Parameters of gut inflammation in WT and TNF^ΔARE^ mice. **(A)** Absolute weight of WT and TNF^ΔARE^ mice during 4 weeks air or CS exposure. **(B)** Lipocalin-2 (LCN-2) protein levels in the faeces of WT and TNF^ΔARE^ mice, as a marker for intestinal inflammation. **(C)** Combined acute and chronic inflammation score in the terminal ileum of WT and TNF^ΔARE^ mice upon 2 weeks air or CS exposure. **(D)** Combined acute and chronic inflammation score in the terminal ileum of WT and TNF^ΔARE^ mice upon 4 weeks air or CS exposure. Histology of the ileum in **(E)** WT and **(F)** TNF^ΔARE^ mice upon 2 weeks of air- or CS-exposure. Values are expressed as mean ± SEM. * p<0.05, ** p<0.01, *** p<0.001.

The clinical appearance of TNF^ΔARE^ mice, which were exposed to air or CS was evaluated. TNF^ΔARE^ mice developed ileitis as assessed by body weight loss (Fig 3A). At the start of CS exposure (defined as day 0), TNF^ΔARE^ mice already weighed significantly less (p < 0,0001) than WT mice, due to the development of ileitis. The mean weight difference between the WT and the TNF^ΔARE^ mice was 3,88 ± 0,53 g. No weight differences were observed between air- and CS-exposed TNF^ΔARE^ mice. In addition, the level of faecal lipocalin-2 (LCN-2), a marker for both histopathologically evident and sub-clinical gut inflammation, was assessed [37]. Higher levels of faecal LCN-2 in TNF^ΔARE^ mice at day 14 and day 21 were observed; however, no differences between air- and CS-exposed TNF^ΔARE^ mice could be detected (Fig 3B). Histological scoring of the terminal ileum was performed by two independent investigators (intraclass correlation coefficient = 0,885). TNF-α overexpression induced ileitis as evidenced by body weight loss and increased histological inflammation in TNF^ΔARE^ mice (Fig 3A, 3C and 3D). After both 2 and 4 weeks of CS exposure, no further increase in TNF-induced histologic inflammation could be observed (Fig 3C and 3D). An ileal section of a WT and TNF^ΔARE^ mouse is depicted in Fig 3E and 3F respectively.

**Fig 4:**
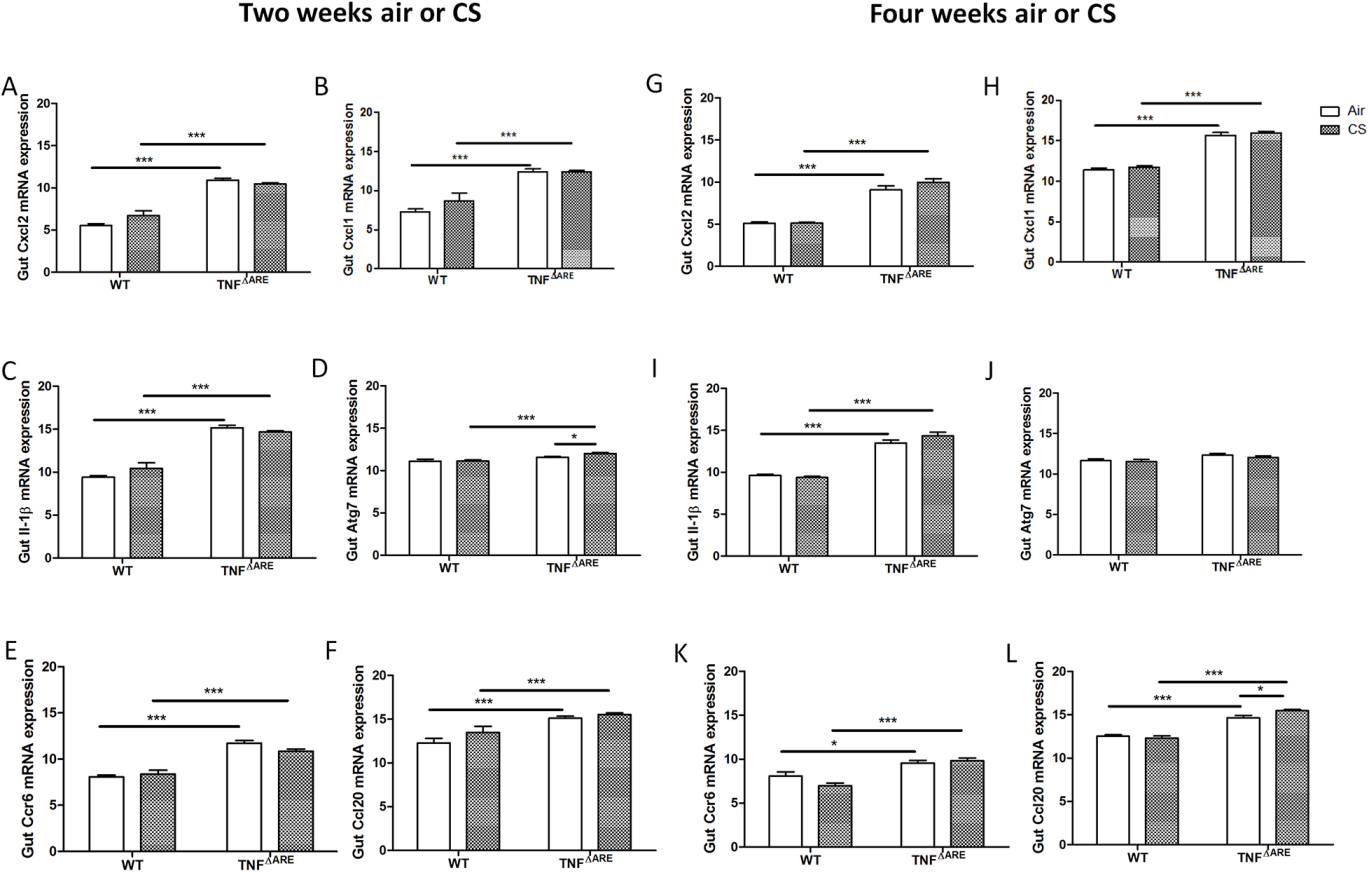
Inflammatory and autophagy gene expression in the ileum of WT and TNF^ΔARE^ mice. mRNA expression of **(A)** Cxcl2, **(B)** Cxcl1, **(C)** Il-1β, **(D)** Atg7, **(E)** Ccl20 and **(F)** Ccr6 in the ileum after 2 weeks of air or CS exposure. mRNA expression of **(G)** Cxcl2, **(H)** Kc, **(I)** Il-1β, **(J)** Atg7, **(K)** Ccl20 and **(L)** Ccr6 in the ileum after 4 weeks of air or CS exposure. Expression levels are relative to the expression of two reference genes (glyceraldehyde-3-phosphate dehydrogenase (GAPDH) and hydroxymethylbilane synthase (HMBS)). Data are expressed as mean ± SEM. * p<0.05, ** p<0.01, *** p<0.001.

In addition, we examined the expression of the cytokines and chemokines, which are upregulated in the ileum by CS exposure [42], namely CXCL2, KC and IL-1β. These factors were elevated in TNF^ΔARE^ mice, however, no further increase was observed after 2 or 4 weeks of CS exposure (Fig 4A–4C and 4G–4I). Furthermore, no major changes in autophagy gene expression were detected (Fig 4D). Thirdly, mRNA expression of the chemokine CCL20 and the chemokine receptor CCR6 was probed, as this is a prominent chemokine-chemokine receptor axis implicated in the response of Peyer’s patches to CS exposure [41]. CCL20 and CCR6 expression was significantly induced in the ileum of TNF^ΔARE^ mice (Fig 4E, 4F, 4K and 4L). However, CCL20 protein remained unchanged (data not shown). In addition, 4 weeks of CS exposure showed a very slight increase in TNF-induced expression of CCL20 (Fig 4). As expected, mRNA expression of TNF-α was elevated in TNF^ΔARE^ mice compared to WT mice. However, nu further increase in TNF-α has been found due to CS exposure (data not shown).

### Short term CS exposure does not affect clinical score and histology in the joints

**Fig 5:**
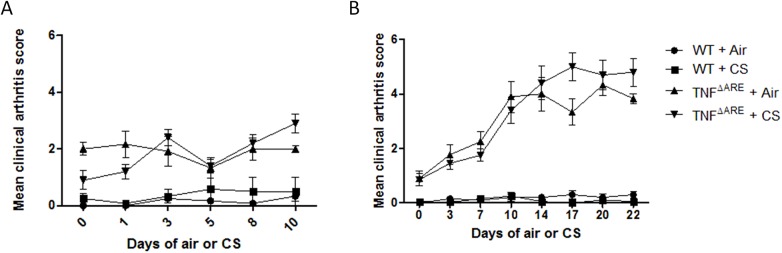
Mean clinical arthritis score in WT and TNF^ΔARE^ mice. Clinical arthritis score during the course of **(A)** 2 weeks (WT vs. TNF^ΔARE^: p < 0,005) and **(B)** 4 weeks CS exposure (WT vs. TNF^ΔARE^: p < 0,005).

**Fig 6:**
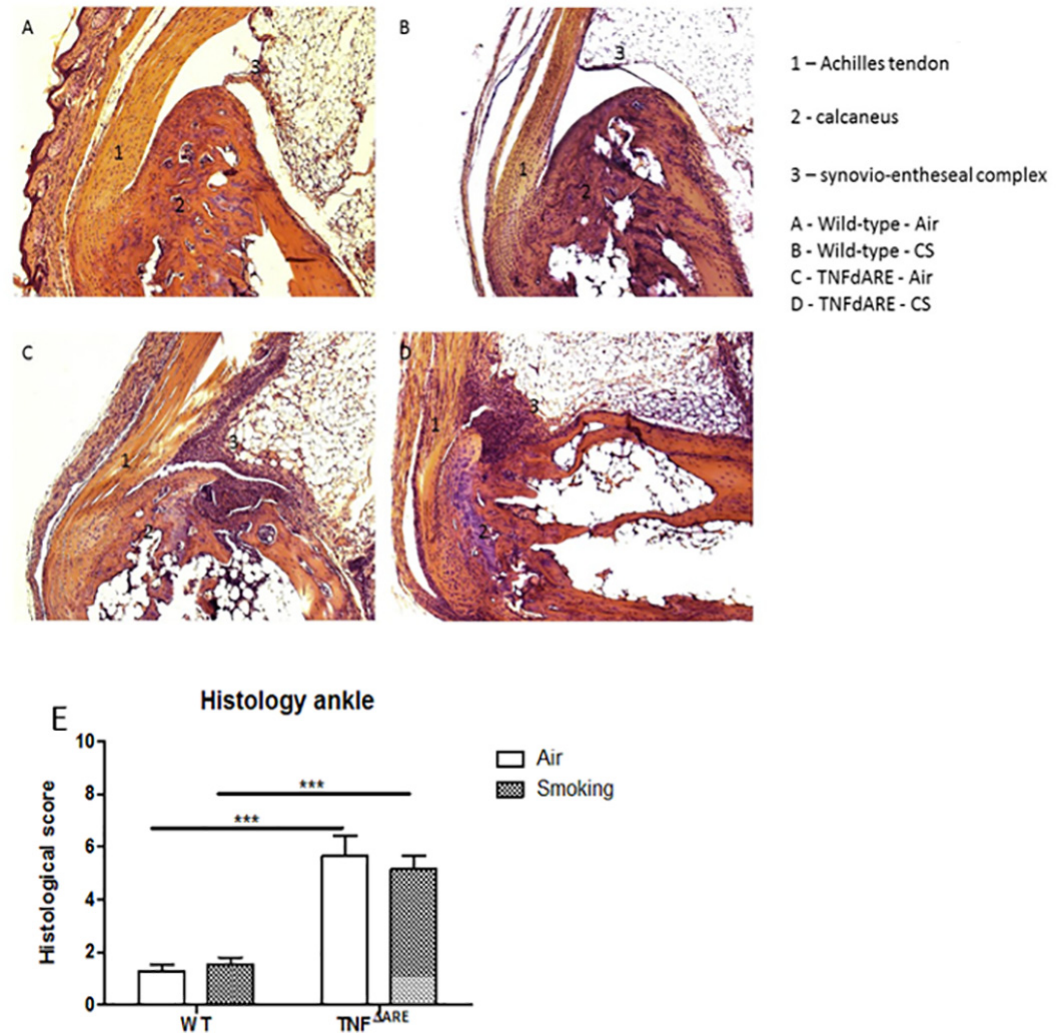
Histology of the joints in WT and TNF^ΔARE^ mice. H&E staining of the ankle joints of **(A)** air-exposed WT mice, **(B)** CS-exposed WT mice, **(C)** air-exposed TNF^ΔARE^ mice and **(D)** CS-exposed TNF^ΔARE^ mice. **(E)** Histological score of inflammation in the joints of WT and TNF^ΔARE^ mice. Histological inflammation is increased in TNF^ΔARE^ mice compared to WT mice (p < 0,005). Data are expressed as mean ± SEM. * p<0.05, ** p<0.01, *** p<0.001.

The degree of arthritis was clinically evaluated at the tarsal and metatarsal region of both WT and TNF^ΔARE^ mice during the 2 and 4 weeks CS exposure experiment. In both experiments, the mean clinical arthritis score was significantly different between the WT and TNF^ΔARE^ group. However, within the WT and TNF^ΔARE^ group, no significant increase in arthritis score due to short term CS exposure could be observed (Fig 5). These clinical observations were confirmed by the histology results. The total histological score, and thus the amount of inflammation and tissue destruction in the joints was clearly higher in the TNF^ΔARE^ group compared to the WT group. The largest difference between these groups was seen in the amount of cellular infiltrate at the synovio-entheseal complex in the tarsal joints. However, no significant differences were observed upon CS-exposure in both groups (Fig 6). Knee joints were also histologically examined, but no significant difference was observed between TNF^ΔARE^ and WT mice (data not shown).

### CS exposure does not affect TNF-α serum levels

**Fig 7:**
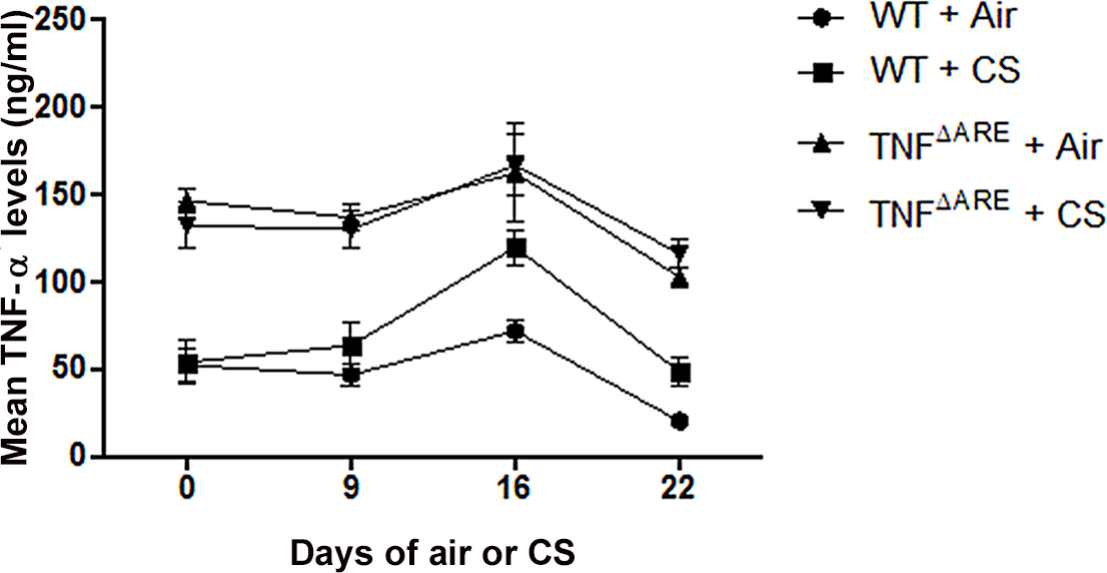
Mean TNF-α levels in serum of WT and TNF^ΔARE^ mice. Protein levels of TNF-α in serum of WT and TNF^ΔARE^ mice throughout the 4 weeks air- or CS-exposure. As expected, TNF-α was increased in TNF^ΔARE^ mice compared to WT mice (p < 0,005).

ELISA for TNF-α was performed on serum at different time points in the 4 weeks CS exposure experiment. As expected, a significant difference in TNF-α levels was observed between the WT and TNF^ΔARE^ mice. However, there was no difference in TNF-α-levels between air- and CS-exposed mice (Fig 7).

## Discussion

This is the first study to report the effects of CS on multiple organ systems (lung, gut and joints) in TNF^ΔARE^ mice as a model of combined pathologies. In the lungs of TNF^ΔARE^ mice, inflammation was aggravated after 2 weeks of CS exposure. In contrast, after 4 weeks of CS exposure, this aggravation was abolished with even a decrease in neutrophils, CD4+ and CD8+ T cells. In the gut and joints of TNF^ΔARE^ mice, 2 or 4 weeks of CS exposure did not modulate the development of gut and joint inflammation. Although the effects of TNF-α overexpression in the lungs have been described before, CS-induced pulmonary inflammation within the context of TNF-α overexpression has not yet been studied [43,44,45].

In the lungs of TNF^ΔARE^ mice exposed to 4 weeks of air, which is at the age of 10 weeks, we denoted an increase in neutrophils, dendritic cells, CD4+ and CD8+ T cells. This response was not found in TNF^ΔARE^ mice exposed to air for 2 weeks, at the age of 8 weeks. In line with studies showing the importance of TNF-α and its receptors in CS-induced pulmonary inflammation [46,47], we observed an aggravation of inflammation in the lungs and a simultaneous increase in inflammatory gene expression levels in TNF^ΔARE^ mice upon 2 weeks of CS exposure. However, after 4 weeks of CS exposure, the inflammation was dampened in the TNF^ΔARE^ mice, with decreased numbers of inflammatory cells in BAL fluid despite the still increased inflammatory gene expression. This temporal difference in inflammatory reaction may reflect an anti-inflammatory state observed during critical illness and far progressed disease states [48] or a tolerance effect induced by dampening of the immune response [49]. Interestingly, in the 2 weeks experiment, there is no difference in the expression of TNFR2 between CS-exposed WT and TNF^ΔARE^ mice. In contrast, in the 4 weeks experiment the expression of TNFR2 is significantly higher in CS-exposed TNF^ΔARE^ mice, compared to CS-exposed WT mice. Most of the inflammatory responses attributed to TNF-α and T cell apoptosis occur through TNFR1, which is the primary signaling receptor. In contrast, TNFR2 directs TNF-α-mediated T cell proliferation, in addition to its antagonizing function through TNF-α neutralization [50]. Furthermore, TNFR have been shown to play a role in COPD [51]. Taken together, the higher TNFR2 expression levels and the loss of the aggravated inflammatory response in the lungs of TNF^ΔARE^ mice upon 4 weeks CS exposure suggest that this receptor possibly modulates the inflammatory response.

In CD patients, cigarette smoking has detrimental effects on the clinical course of disease, necessitating increased need for steroids, immunosuppressive drugs, and surgery [31,32]. TNF-α is known to be aberrantly overproduced in CD and anti-TNF is commonly used as a treatment [9]. It is likely that CS acts as a predisposing factor for CD development in susceptible individuals, as we previously demonstrated in an experimental model of CS exposure in healthy mice [41,42,52]. In the dextrane sodium sulphate (DSS)-induced colitis mouse model showing features of human ulcerative colitis (UC), it has been shown that TNF-α is decreased in the colon due to CS exposure [53]. UC is known to be mainly a disease of ex-smokers. We chose the TNF^ΔARE^ model for our study as its deletion in the AU-rich elements of the TNF gene is ubiquitous and therefore combines multiple TNF-mediated pathologies such as CD and SpA. Also, this model shows Crohn-like ileitis, which can only be obtained with an altered genetic context. Th1-driven CD8+ T cells play a dominant role in the development of ileitis in the TNF^ΔARE^ model [40]. Since we previously demonstrated that chronic smoke exposure elicits the recruitment of the CD8+ T cell subset to the Peyer’s patches of healthy mice [41], this seemed a great model to investigate CS-induced effects on ileitis.

Epidemiologic studies have shown that cigarette smoking influences radiographic progression, quality of life and occurrence of comorbidities in SpA-patients, more specifically ankylosing spondylitis [54,55]. The mechanisms underlying this phenomenon have not yet been investigated in SpA, in contrast to RA. The TNF^ΔARE^ mouse is an interesting SpA-like model to study the effect of CS exposure on disease initiation and progression, since the development of arthritis in these mice does not depend on T- and B-cells, and thus the production of auto-antibodies, as shown by Kontoyiannis et al [34].

The TNF-α overproduction in the TNF^ΔARE^ mice resulted into ileal inflammation, with a decrease in body weight, an increase in LCN-2, histological inflammation and inflammatory gene expression, which confirms previous studies [34,40]. The same was observed in the joints, with an increase in clinical arthritis score and histological inflammation in the ankles [27]. Also, an increase in TNF-α levels was observed in serum of both air- and CS-exposed TNF^ΔARE^ mice. The effects of 2 and 4 week CS exposure on the gut and joints of TNF^ΔARE^ mice were examined. Histological inflammation in the ileum and ankle and knee joints was not aggravated by this short term CS exposure in WT or in TNF^ΔARE^ mice. Also, TNF-α were not elevated in serum of CS-exposed TNF^ΔARE^ mice compared to air-exposed TNF^ΔARE^ mice. Only in the ileum, minor gene expression changes occurred: *Ccl20* mRNA in TNF^ΔARE^ mice, but not CCL20 protein, was increased in TNF^ΔARE^ mice after 4 weeks CS exposure. Despite the minor gene expression changes we detected, we could not show an additional effect of 2 and 4 weeks CS exposure in the ileum or in the joints.

Although CS exposure clearly affects the immune response in the lung, no effect on inflammation in the gut and the joints was found. This might be partly explained by the indirect action of CS on gut and joints, while the lungs are the main contact site for CS. However, in human, CS does affect disease course of CD and SpA. Indeed, humans often encounter pathogens, which might promote the effects of CS, while the mice are kept in a standardized and controlled environment. Also, the CS exposure protocol might be too short or too weak to evaluate the effect of CS in the gut and joints. Moreover, the phenotype of CD- and SpA-like inflammation in TNF^ΔARE^ mice can be too strong to still observe a further aggravation by CS exposure.

In conclusion, our findings demonstrate that the deregulated TNF-α expression in TNF^ΔARE^ mice aggravates CS-induced pulmonary inflammation upon 2 weeks of exposure. Intriguingly, this aggravated response in lungs of TNF^ΔARE^ mice is attenuated upon 4 weeks of CS exposure. The TNF^ΔARE^ phenotype encompasses the development of TNF-α-induced inflammation in the ileum and the joints. However, in the gut and joints of TNF^ΔARE^ mice, CS exposure for 2 or 4 weeks did not alter the TNF-α -induced development of CD and SpA.

## Supporting Information

S1 Dataset Raw data of qPCR, ELISA and histology. (XLSX)
